# 2SpamH: A Two-Stage Pre-Processing Algorithm for Passively Sensed mHealth Data

**DOI:** 10.3390/s24217053

**Published:** 2024-10-31

**Authors:** Hongzhe Zhang, Jihui L. Diaz, Soohyun Kim, Zilong Yu, Yiyuan Wu, Emily Carter, Samprit Banerjee

**Affiliations:** 1 Division of Biostatistics, Department of Population Health Sciences, Weill Cornell Medical College, New York, NY 10065, USA; 2 Weill Cornell Institute of Geriatric Psychiatry, Weill Cornell Medical College, White Plains, NY 10605, USA

**Keywords:** k-nearest neighbors algorithm, mobile health, smartphone, passive sensing

## Abstract

Recent advancements in mobile health (mHealth) technology and the ubiquity of wearable devices and smartphones have expanded a market for digital health and have emerged as innovative tools for data collection on individualized behavior. Heterogeneous levels of device usage across users and across days within a single user may result in different degrees of underestimation in passive sensing data, subsequently introducing biases if analyzed without addressing this issue. In this work, we propose an unsupervised 2-Stage Pre-processing Algorithm for Passively Sensed mHealth Data (2SpamH) algorithm that uses device usage variables to infer the quality of passive sensing data from mobile devices. This article provides a series of simulation studies to show the utility of the proposed algorithm compared to existing methods. Application to a real clinical dataset is also illustrated.

## 1. Introduction

### 1.1. Background

The market for digital health technologies has expanded due to recent advancements in mobile health (mHealth) technology and the ubiquity of smartphones. mHealth devices can be classified into two broad classes based on their intended use, i.e., research-grade devices and commercially available devices. Research-grade devices typically have sensors designed to collect uninterrupted measurements under the supervision of researchers, often in a laboratory setting. In contrast, commercially available wearable devices and smartphones, with sensors designed to collect measurements intermittently, are commonly used in everyday life in users’ natural environments without supervision. Because commercially available devices are used in the users’ natural environment (as opposed to a laboratory setting), data from smartphones and wearables have potential for assessing and monitoring mental and behavioral health. In addition, digital interventions delivered via commercial devices have broader reach due to the ubiquity of these devices. Consequently, these commercially available devices have emerged as innovative tools for monitoring disease status and early detection of symptoms of a disease, as well as delivering digital interventions [1].

Commercial mHealth devices (e.g., smartphones, wearables) passively record data from their sensors such as GPS (location coordinates i.e., longitudes, latitudes), microphone, and accelerometer (e.g., three-dimensional accelerations), without any user input. These “raw” data from the sensors are used to derive or infer behavioral features, such as physical activity level [2], the number of places visited [3], the amount of time spent in conversation [2], and the quality and duration of sleep [4,5]. They provide insight on users’ physical activity, sleep patterns, and sociability. The focus of this paper is on passive data collected from commercial mHealth devices, and from here on mobile devices or devices refer to commercial mHealth devices unless mentioned otherwise.

### 1.2. Downward Bias in Passive Sensing Data

Due to multiple reasons, including privacy and the optimization of CPU usage and battery life, mobile devices employ strategies to record passive data periodically rather than continuously [3]. For example, real-time audio samples (non-speech content) from the smartphone’s microphone are recorded only at regular intervals [6]. Similarly, GPS tracking platforms use adaptive duty-cycling algorithms designed to conserve energy that periodically turn sensors on (active phase) and off (sleep or idle phase) [7]. The duration of sleep or an idle phase is adapted based on the events detected during the active phase. For example, for a microphone, if the active phase detects voice, then the consequent pre-programmed duration of the sensor’s sleep phase is reduced or vice versa [8,9]. The recorded data are only uploaded or synced with the cloud occasionally after being aggregated over a time interval (e.g., hourly, daily) [10]. The frequency of these uploads/syncs is adjusted in real-time based on factors such as the storage capacity of the device, battery level, and network connectivity. However, the algorithms governing these decisions to activate sensors and upload data are often black-box and proprietary for commercial devices; thus, we cannot infer any systematic relationship between individual device usage and sensor activity. These strategies are designed to optimize battery life and manage demand on network resources since data are stored locally on the device for a certain period before being transmitted to the cloud in batches.

**Figure 1:**
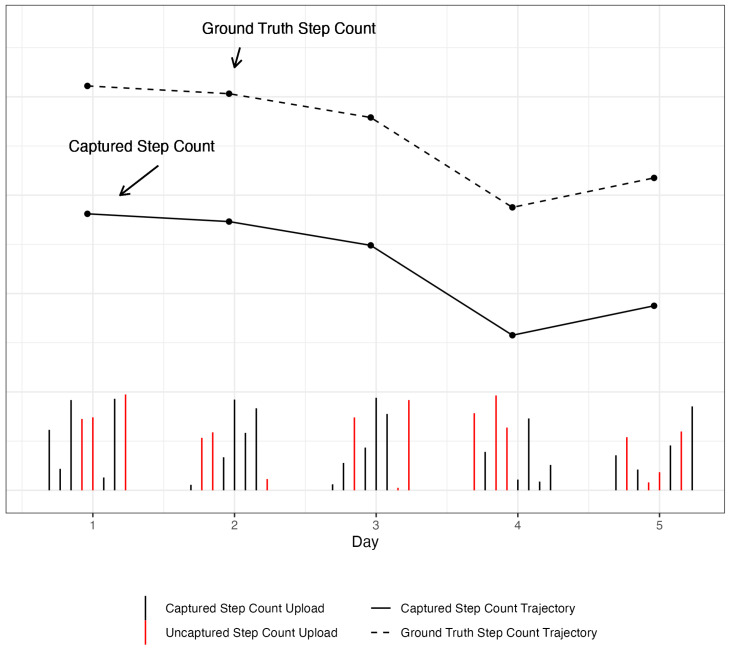
A simulated example of downward bias introduced by duty-cycling algorithm. The vertical black and red lines represent the captured and uncaptured uploads of the true raw sensor data, respectively. The uncaptured uploads represent activity that is uncaptured by the sensor either because the sensor is off (due to duty-cycling) or the device is not being used or worn by the user. Each point, a daily aggregated step count, is derived as the sum of all uploads of raw sensor data from that day (black vertical lines), while the daily hypothetical upload is the sum of both the captured uploads (black vertical lines) and uncaptured uploads (red vertical lines) from that day. The upper trajectory of the dashed black line represents the trajectory of the true step count uploads while the lower trajectory of the solid line is the observed trajectory of captured step count uploads, an underestimation of the ground truth trajectory.

Although these strategies help to optimize battery life and minimize CPU usage, thereby improving device performance and allowing realistically sustainable data collection, they intrinsically introduce a downward bias to the passive sensing data. For example, among all voice events in a day (i.e., phone conversations as a measure of sociability), some may be missed because they occurred during an off cycle of the microphone sensor, i.e., the sensor was turned off due to the duty-cycling. Therefore, a systematic downward bias is introduced in the recording of passive measures [6]. However, a reasonable assumption can be made that the degree of downward bias does not vary with time for a user, as long as the user uses the same device. To illustrate the downward bias introduced by duty-cycling algorithms on passive sensing data collection, a simulated example is presented in Figure 1. In this hypothetical example, the ground truth trajectory of step count, unknown in real-world scenarios, is the dashed line and the trajectory of the step count captured by the device is the solid black line; the parallel downward shift of the captured trajectory relative to the ground truth trajectory demonstrates that the user-specific trend over time is preserved despite the downward bias present in passive measures. While “true” values of the passive measures may be underestimated due to this downward bias based on this assumption, within-person trends over time may still provide meaningful insights into user behavior for research purposes [6].

An additional source of downward bias in passively sensed data is introduced due to the unknown device use by the user. Unlike research-grade devices which record data in a laboratory or supervised setting where the device use or wear status is known, passive data from commercial devices record data in the wild where the device use or wear status is unknown. Passive data are only recorded if the user carries their smartphone or wears the wearable device, but it is not known when they are not using or wearing the device [1,11]. This unknown device non-use or non-wear status may not be consistent for a user over time. Therefore, it is necessary to adequately correct for device non-use or non-wear before using passive data from commercial devices to gain meaningful within-person changes over time [12]. This paper focuses on the development of a pre-processing algorithm that addresses the downward bias introduced from unknown device non-use or non-wear by identifying observations we infer to have significant non-use or non-wear (i.e., missing).

### 1.3. Existing Methods for Evaluating Sensor Data Validity

In the literature, the problem of downward bias in raw sensor data is referred to as “sensor data validation” and has been studied outside of the field of mobile health. Previous data validation methods include simple numerical checks to examine minimum and maximum values [1] and physical and logical data ranges [5]. Other studies define the sensors as a measurement system in the presence of noise and aim to declare the sensor faulty when it has unreliable data [13]. In another study, the authors create training data with validity labels obtained from reported wear time and train a support vector machine classifier with engineered features representing activity measures [14]. However, such training data may not be available to every study involving an mHealth device. Except for simple numeric checks, other methods typically require supervision (i.e., known device use or wear status) to a certain degree (i.e., either entirely supervised or at least a supervised training data are available), whereas commercially available devices, unlike research-grade devices, are typically unsupervised (i.e., unknown device use or wear status). In mobile health, researchers typically perform ad-hoc analysis and apply arbitrary thresholds to the data recorded by the mobile device on a specific time interval (e.g., a day) to determine non-use or non-wear. These thresholds, which are constant across users, are not personalized and therefore unsuitable even for a group of relatively homogeneous individuals in a clinical study [14,15]. For example, a threshold of 500 step-counts or below in day used to determine device non-use or non-wear may be appropriate for an older adult with functional impairment but may not be appropriate for a younger active individual (e.g., a runner). One more recent approach, proposed by Kraft et al. integrates Ecological Momentary Assessments (EMAs) and Principal Components Analysis (PCA) to detect sensor anomalies in environmental sound levels with a mobile health crowdsensing platform [16]. The algorithm uses EMA data to inform its analytic approach to correct sensor errors. However, this method requires supervision from the user in the form of concurrent EMA, which may not always be available. Our method is designed to be more widely applicable as it only requires passively sensed data collected by devices such as smartphones and wearables.

### 1.4. Our Solution

We view the unknown device non-use or non-wear as a statistical missing data problem, where the missing data labels are also unknown. To this end, we propose an unsupervised algorithm called 2-Stage Pre-processing Algorithm for Passively Sensed mHealth Data (2SpamH) that predicts these missing data labels using auxiliary device usage data and some reasonable assumptions. We begin by discretizing summaries of a passive measure for a particular user based on pre-defined time intervals, e.g., total step count in a day. Then, 2SpamH follows two stages—in the first stage, it converts an unsupervised problem (unknown device use or wear) to a semi-supervised problem by using auxiliary information about engagement with the device (e.g., screen unlock events, battery use, etc.) as a proxy for device use to define prototypes where device use or wear status can be assumed to be known with some confidence; in the second stage, using the prototypes in the first stage, the device use or wear status is inferred on observations where there is uncertainty of their use or wear status.

2SpamH is applicable to any passive measure (e.g., step counts, hours of sleep, etc.); however, we focus on the daily step count as an example in this paper. We conducted a series of simulation studies to demonstrate the performance of 2SpamH. We also apply 2SpamH to the smartphone data collected from elderly patients with major depressive disorder by Weill Cornell Advanced Laboratory for Accelerating the Reach and Impact of Treatment for Mid- and Late-Life Depression (ALACRITY) Research Center (P50MH113838). We conclude by demonstrating statistical imputation strategies to impute data deemed as “missing” data by 2SpamH due to device non-use or non-wear. The imputed dataset can subsequently be used for downstream analysis of user behavior in various healthcare applications.

## 2. Materials and Methods

The goal of 2SpamH is to address the downward bias (described in Section 1.2) introduced by unknown device non-use or non-wear. Since device use or wear behavior over time is user-specific, 2SpamH is performed one user at a time. Also, passive measures (e.g., step counts, hours of sleep, etc.) can be derived from one or multiple sensors and 2SpamH is applied separately for each passive measure. The central idea of the 2SpamH algorithm is to utilize readily available auxiliary device engagement information (e.g., number of screen unlocks, battery variance, etc.) as a proxy of device use and combine it with sensor activity (as a proxy of data from a sensor being recorded) to estimate the device non-use status. Below, we define the data, algorithm, and its key assumptions.

### 2.1. Notation and Specifications

The data on passive measures collected from a user’s device are first discretized into pre-determined time intervals (e.g., daily summaries) and these summaries (e.g., total step counts in a day) are stored in a matrix $X^{i}$ of passive variables, where each column represents a passive variable (e.g., step count) and the superscript *i* represents each user. The number of rows represents the number of time intervals (e.g., number of days) observed for a particular user and may vary across users, but the number of columns, representing the passive variables, is constant as we assume that devices collect the same variables across users. The sensor activity corresponding to a passive variable of user *i* is stored in matrix $W^{i}$. Sensor activity is a proxy of the data being recorded by the sensors that are used to infer a particular passive variable and are operationalized by the number of uploads (more details in Section 2.2). In Figure 1, these are the number of uploads (black vertical ticks) for a specific day. Of note, the matrices $X^{i}$ and $W^{i}$ have the same number of rows since for a specific day, an aggregated step count measure and the total number of uploads are uploaded to the server. The matrix $Z^{i}$ stores all variables related to device usage; the number of rows of $Z^{i}$ is also the same as that of $X^{i}$ and $W^{i}$. Finally, the output of the 2SpamH algorithm is a matrix $M^{i}$, of the same dimension as that of $X^{i}$, that has a binary label (“missing” or “non-missing”) indicating whether a particular cell of the matrix (i.e., a passive variable for a day) should be considered “missing” for downstream analysis. For easier readability, we ignore superscript *i* from here on and assume that all data structures are user-specific.

### 2.2. Stage 1 of 2SpamH: Feature Space Construction via Principal Component Analysis and Prototype Selection

**Figure 2:**
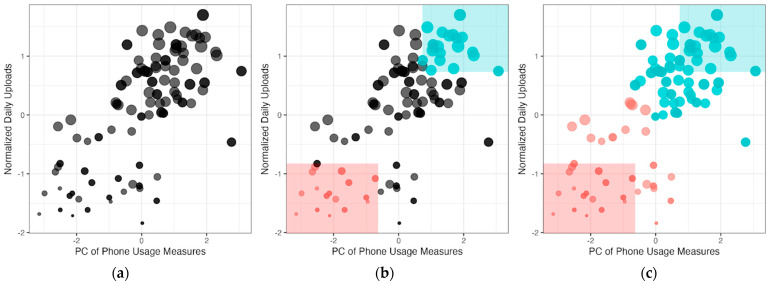
Step-by-step illustration of the 2SpamH algorithm, where the size of each data point on the constructed feature space represents a daily observation of step count; (**a**) feature space construction with the first principal component of phone usage measures (*x*-axis), and the normalized number of step count uploads (*y*-axis); (**b**) prototype selection (red = “missing”, blue = “non-missing”); (**c**) k-nearest neighbors algorithm.

**Figure 3:**
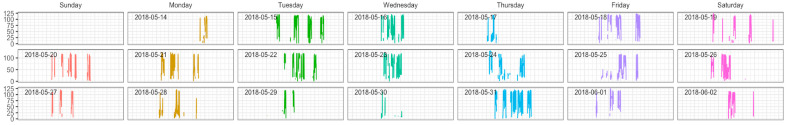
Daily upload of step counts from the device of a single user over a three-week period, colored by the day of the week.

To estimate the matrix M of the missing status, 2SpamH, in its first stage, utilizes auxiliary information readily available from commercial devices (i.e., Z and W) that provides implicit information on device-use status and informs a more personalized data pre-processing algorithm. For example, if a device has not been unlocked or moved for a prolonged period, the data it collected during that period are unlikely to be representative of the user’s actual step counts; if the battery variance is low, it is likely that the device is constantly being charged or out of charge and device use is low. The number of screen unlock events and battery variance are typically available from smartphone apps. Wearable devices also have similar auxiliary information (e.g., wear time) which can be used for this purpose. The key concept here is to identify auxiliary variables that correlate with device use (e.g., screen unlocks and battery variance for smartphones and wear time for wearables). In the event there are multiple variables, such as in typical smartphone apps, that correlate with device usage, we recommend conducting a principal component analysis or PCA on these variables and using the first principal component as a device use measure. Of note, if there are multiple variables for device use, they may need to be recoded in a way that the first principal component indicates high device use. Next, a feature space is constructed that represents device usage (or PCA of device usage) in one dimension and sensor activity in another dimension. This is illustrated in Figure 2a, a scatter plot of a passive variable (one column of the matrix X), where the size of each observation is proportional to the daily step count. Sensory activity is operationalized as the number of uploads to the server. Note that the number of uploads is a proxy of sensor activity assuming that when a sensor is active and data are recorded, data are more frequently uploaded to the server (e.g., see Figure 3 and Section 1.2). Figure 3 shows the recorded step count of a user for three weeks and demonstrates that the number of uploads (spikes in the data) varies widely across different days and weeks, highlighting possible variations in the sensor activity of a user.

It should be noted that the output of 2SpamH is the matrix M that determines the device non-use or non-wear status (i.e., considered “missing” for downstream analysis) for each day and passive variable for a user. In that sense, Stage 1 is an unsupervised problem as the “missing” labels are absent. Next, we assume that certain regions in this feature space (Figure 2) can be considered as “missing” or “non-missing” with some confidence and consider data points falling in these regions as prototypes for inferring the missing status of data points outside this region. “Data point” refers to coordinates on the feature space of the principal component of device usage (*x*-axis) and number of uploads (*y*-axis) of Figure 2, corresponding to the *t*th observation in $Z^{i}$ and $W^{i}$. This is denoted as $f_{t}$ in the pseudocode (Algorithm 1). This assumption is a key step as the unsupervised problem of unknown “missing” labels is transformed to a semi-supervised problem where the “missing” labels are partially observed in Stage 2 via this assumption. Table 1 illustrates the conceptual framework behind this assumption. A lower ${(\theta }_{lower})$ and upper ($\theta_{upper})$ threshold is assumed in each dimension of the feature space to define “low” and “high” levels in each dimension of device usage and sensor activity. When both device usage and sensory activity are “low”, we assume device use or wear is low and label the data point as “missing”. Similarly, when both device usage and sensory activity are “high” we assume that device use or wear is high and therefore label the data point as “non-missing”. This is illustrated in Figure 2b where the data points in the red colored region are considered “missing” prototypes and those in the blue colored region are considered “non-missing” prototypes. On the other hand, data points with high device usage with low sensor activity and low device usage with high sensor activity are more challenging to label. For example, when device usage levels are high but sensor activity is low, the user frequently unlocks and interacts with their phone throughout the day, but the sensor activity or number of uploads are infrequent; it is uncertain whether a low number of uploads accurately reflects the user’s true activity level (which may be high and not captured because the device usage period was distinct from the activity period when the device was not carried/worn) or if they were simply inactive. On the other hand, when device usage levels are low, but the number of uploads is high, the user rarely interacts with his or her phone, but the sensor data uploads are frequent showing high sensor activity due to sensor errors. As an example, a microphone sensor inferring time spent in interpersonal conversation as a passive measure may erroneously misclassify a television conversation as an interpersonal conversation [17].
**Algorithm 1.** Pseudocode of the 2SpamH algorithm which has two stages: (1) prototype selection in the feature space of device use and sensor activity levels to label data points as “missing” or “non-missing” with some confidence based on a threshold, and (2) a k-nearest neighbors (KNN) approach to label non-prototype data points in the feature space based on their proximity to the labeled prototypes. The algorithm returns “missing” labels for all data points.**2SpamH Algorithm****Input:** Sensor activity matrix W, Device usage matrix Z, Prototype selection percentiles $\left\{\theta_{lower},\;\theta_{upper}\right\}$, Number of nearest neighbors k
**Output:** Missing label matrix M
**Stage 1: Prototype Selection**1.Perform PCA on Z and W to obtain the principal components:
$C^{Z}=PCA(Z),\;C^{W}=PCA(W)$
where $C^{Z}$ and $C^{W}$ are vectors of length *T* of the first principal components of Z and W. If ncol(Z) = 1, then $C^{Z}=Z$; if ncol(W) = 1, then $C^{W}$= W.2.Construct the feature space F as the set of points $f_{t}=(C_{t}^{Z},C_{t}^{W})$ for each *t*:
$F=\{f_{t}|t=1,\;\ldots ,\;T\}$
where $f_{t}=(C_{t}^{Z},C_{t}^{W})$ represents the coordinates of the *t*^th^ data point in the constructed feature space.3.Compute the lower and upper quantiles for $C^{Z}$ and $C^{W}$:
$q_{lower}^{Z},q_{upper}^{Z}=Q(C^{Z},\;\theta_{lower}),Q(C^{Z},\;\theta_{upper})$
$q_{lower}^{W},q_{upper}^{W}=Q(C^{W},\;\theta_{lower}),Q(C^{W},\;\theta_{upper})$4.Identify the set of missing prototypes in the feature space F:
$P_{missing}=\{f_{t}\in F|C_{t}^{Z}<q_{lower}^{Z}\;and\;C_{t}^{W}<q_{lower}^{W}\}$5.Identify the set of non-missing prototypes in the feature space F:
$P_{non\text{-}missing}=\{f_{t}\in F|C_{t}^{Z}>q_{upper}^{Z}\;and\;C_{t}^{W}<q_{upper}^{W}\}$6.For each data point $f_{t}\in F$**:**7.Assign labels to rows of M based on whether data points fall within the prototype regions:
$M_{t,:}=\left\{\begin{array}{cc} Missing & \;if\;f_{t}\in P_{missing}\\ \mathrm{Non}\text{-}\mathrm{missing} & if\;f_{t}\in P_{non\text{-}missing}\\ NA & \;otherwise \end{array}\right.$**Stage 2: Labeling Unlabeled Data Using KNN**8.For each unlabeled data point $f_{t^{\prime }}\in F$ that was not assigned a label in Stage 1:9.Implement KNN with K=k and Euclidean distance function           $d\left(f_{t},\;f_{t^{\prime }}\right)=\sqrt{\left({\left(C_{t}^{Z}-{C_{t}^{Z}}^{\prime }\right)}^{2}+{\left(C_{t}^{W}-{C_{t}^{W}}^{\prime }\right)}^{2}\right)}$ to label the remaining unlabeled data points:           $M_{t^{\prime },:}:=KNN(f_{t^{\prime }}|P_{missing},\;P_{non\text{-}missing},\;k)$
**Return:** Missing label matrix M.


### 2.3. Stage 2 of 2SpamH: K-Nearest Neighbors Algorithm

Once the prototypes are selected, they serve as a set of labeled training data with which the k-nearest neighbors (KNN) or any standard supervised learning procedure can be applied [18]. A user-specific classifier is trained on the prototypes (colored points in Figure 2b) and is used to predict the remaining unlabeled data points (black points in Figure 2b) using the majority vote of the labels of the nearest *k* prototypes, i.e., *k* is the number of neighbors. This completes the 2SpamH algorithm where the missing label matrix M is estimated for each data point (Figure 2c). For all users, we perform the steps described above in Section 2.2 (Stage 1) and Section 2.3 (Stage 2) and outlined in Algorithm 1 below. 

### 2.4. Remarks on Parameter Tuning

The thresholds used in the prototype selection step (i.e., $\theta_{lower}$ and $\theta_{upper}$) and the number of neighbors (*k*) are left undetermined. In addition, the KNN algorithm in Stage 2 of 2SpamH can be implemented with different distance functions and classification rules. As the true missing labels are unknown, common methods for parameter tuning such as cross-validation cannot be applied.

The choice of the thresholds $\left(\theta_{lower},\theta_{upper}\right)$ is dependent on the context and the data. We used the 30th and 70th quantile as the lower and upper thresholds in our simulation study and real-data application. We also demonstrate the effect of different choices in our simulation study (Supplementary Figure S1) and in real data (Supplementary Figure S2). A practitioner may choose these thresholds based on the number of prototypes selected for each of the two labels, i.e., “missing” and “non-missing” since using quantiles as thresholds in each dimension of the feature space (Figure 2) separately does not guarantee enough prototypes in the two-dimensional feature space. As an alternative, one may also consider bivariate quantiles or contours [19,20].

The neighborhood size *k* of the KNN depends on the number of observations per user (i.e., the number of rows of X) and should not exceed half of this neighborhood size, i.e., *k*/2 should not exceed the number of prototypes labeled as “non-missing” or “missing” (to maintain class balance in the neighborhood). If this condition is not met, there would not be enough labeled prototypes to form a reliable basis for classification and all non-prototype observations would be labeled by the majority class of the prototypes. For example, consider a dataset with 20 prototypes, where 16 are labeled as “non-missing” and 4 as “missing”. If *k* is set to 18, *k*/2 (9) exceeds the number of “missing” prototypes (4). In this case, the neighborhood would always contain more “non-missing” prototypes, biasing the classification towards the majority class.

We used the Euclidian distance function in our KNN implementation. Alternative distance functions, such as Mahalanobis distance or density-based methods, can also be used, but require additional parameters that would need to be tuned. Tuning such parameters may be challenging in the real-world application of this unsupervised algorithm. Other methods, such as correlation as a distance function, may not be suitable since a pair of observations that have high correlation will be close to each other and thus assigned the same label, when in fact, data points that are close to each other can belong to either class—missing or non-missing. For example, in Figure 2, all points on the 45-degree line are close to each other but include labeled observations in both classes.

As an alternative to the majority voting as the classification rule in the KNN algorithm, the frequency of prototype labels could be a prior distribution to obtain the posterior probability of new unlabeled data points and an alternative voting rule as defined in Coomans and Massart [21].

### 2.5. Simulation Studies

To evaluate the performance of the proposed algorithm, we conducted a simulation study using step count data. Data are simulated for 36 users, with each user assigned a ground truth (known) label of “missing” or “non-missing” to reflect his or her device use for 100 days. The ground truth labels are controlled by two parameters—the continuous activity level $L_{A}$ and the continuous device usage level $L_{U}$. These parameters ($L_{A}$ and $L_{U}$) are then used to simulate the sensory activity matrix W and the device usage matrix Z. Details of this simulation strategy is provided in the Supplementary Materials. Briefly, the hourly activity and device usage status are determined with $L_{A}$ and $L_{U}$ and generate hourly data on sensor activity and device usage (screen unlocks, battery variance, device notification) that match the time of the day (e.g., morning, afternoon, evening, and night). Finally, hourly data are aggregated to provide daily summaries.

We compare the 2SpamH algorithm with two existing methods: the zero-removal algorithm and one-class SVM, using sensitivity (proportion of correctly identified “non-missing” labels) and specificity (proportion of correctly identified “missing” labels). The zero-removal algorithm directly replaces zero values in the simulated passively sensed variable matrix ***X*** with “missing”. This method mimics the usual practice of using an arbitrary cutoff across all users to exclude the observations (e.g., step counts) that are less than the cutoff from downstream analysis. Although intuitive, this practice disregards the heterogeneity in activity levels and phone usage behaviors across users.

The one-class Support Vector Machine (SVM) is trained on data known to be “non-missing” [22], using the feature in both ***W*** and ***Z***. The algorithm learns the typical distributions of the input features for the “non-missing” days, and it estimates a spherical boundary encompassing the “non-missing” days. New data points are then classified as “non-missing” if they lie inside the boundary and “missing” otherwise. In our simulation, we sample 300 observations (out of 3600 observations of 36 users for 100 days) with “non-missing” labels to train the one-class SVM. The trained model is then used to predict the label of all remaining observations. However, one-class SVM requires labeled “non-missing” data, which are typically not available in real-life mHealth data. For consistency, we employ the same tuning parameters for all simulated subjects.

### 2.6. Imputation Techniques

After applying 2SpamH on each passive variable, i.e., each column of X, the output missing label matrix M is complete. The next step is to convert the cells in X with corresponding cells in M as “missing” to missing. Then, standard off-the-shelf missing data imputation techniques can be used to impute the missing data in X. Of note, the imputation should be performed on the entire passive variable matrix X even though 2SpamH is applied on each column of X one at a time because imputation will be better informed from all passive measures that represent the complete behavioral profile of a user. Furthermore, imputation can be carried out by combing the passive measure matrices across users (i.e., $\left\{X^{i}:i=1,\ldots ,n\right\}$) to borrow information across users. To this end, we implement a missForest [23] algorithm for imputation in two ways: within-user and across users. Another aspect of mHealth data is that passive measures are recorded over time for a user (i.e., each column of X) and hence are correlated. To this end, we adopt a methodology called jomoImpute [24] that takes the correlation induced by the time-dependent nature of the data into account.

MissForest is an algorithm that uses random forests to impute missing values in mixed-type data (continuous or categorical). It starts with mean imputation, then trains a random forest with observed data on a passive variable (e.g., step count) with other passive variables as predictors and uses this trained model to predict the missing step counts. This is iterated over all passive variables until the newly imputed data do not substantially improve over the imputed data in the previous iteration. We performed both within-user missForest, where imputation is carried out in a user-specific way, and across-user missForest, where all users are included in the same imputation model. JomoImpute is a method based on mixed-effects modeling, designed for handling hierarchical or multilevel data with missing values. It works in two steps: first, it fits a regression model using the “non-missing” data, treating the incomplete variables as dependent on complete variables or just an intercept. Then, it applies a Gibbs sampling technique to generate estimates for the missing values based on the relationships found in the first step.

To evaluate the performance of imputation using missForest and jomoImpute, we used real-life smartphone data (see Section 3.2). Since the “missing” label is unknown in real data, we carried out the evaluation in four steps—(1) we applied 2SpamH across 11 passive variables; (2) we assumed data deemed as “non-missing” by 2SpamH as “true” non-missing data or the ground truth; (3) we simulated missing values in the data from Step 2, i.e., those determined “non-missing” by 2SpamH, by turning observed values to missing under two different missing data mechanisms, missing at random (MAR) and missing completely at random (MCAR) [25]; and (4) we compared the imputed values to the ground truth using NMRSE (see below). Of note, in Step 3, MCAR assumes that the probability of missing data is unrelated to both observed and unobserved data, representing a completely random process of data missingness, and MAR assumes that the probability of missing depends only on observed data. We generated about 20% to 25% missing data for each variable under both MAR and MCAR assumptions. It is important to note that these simulated missing data are used only for the purpose of evaluating the performance of imputation on real data.

We then applied jomoImpute using a mixed model, selecting one passive variable at a time as the outcome, and including a user-level random intercept with fixed effects for the other passive, EMA, and demographic variables (see Section 3.2). Imputation accuracy was measured using the normalized rooted mean squared error (NRMSE), with smaller values indicating a better performance. Since the missing values were simulated, we can compute the NRMSE which measures the error between the imputed value and the observed value. The formula for computing the NRMSE from the RMSE (root mean squared error) is

(1) $$NRMSE=\sqrt{\frac{mean{\left(X_{obs}-X_{imp}\right)}^{2}}{var\left(X_{obs}\right)}}$$

where $X_{obs}$ and $X_{imp}$ are the observed and imputed passive measures, respectively.

## 3. Results

### 3.1. Simulation Results

**Figure 4:**
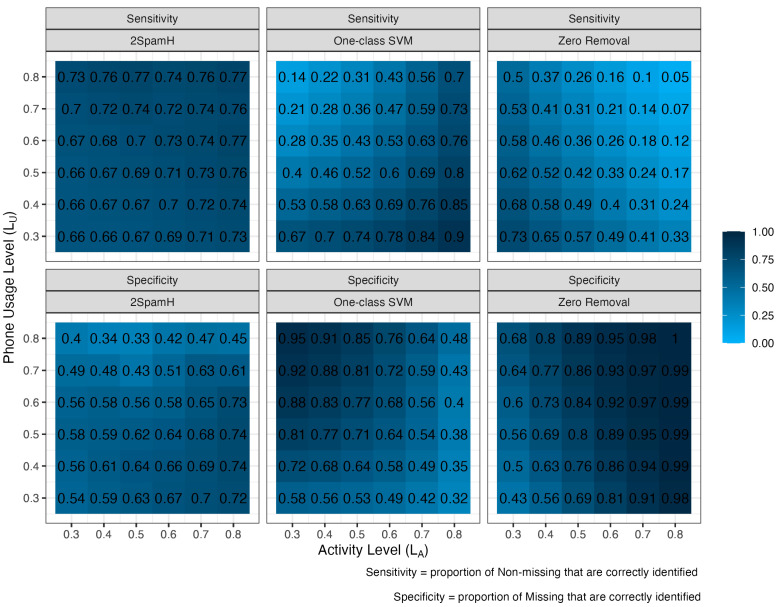
The sensitivity and specificity performances of all three algorithms. Each block in the grids represent the algorithm performances at a specific activity level ($L_{A}$) and phone-usage level ($L_{C}$).

We examine the performance of the 2SpamH algorithm in our simulated data using the 30th and 70th quantiles as lower and upper thresholds (i.e., $\theta_{lower}$ and $\theta_{upper}$ in Stage 1 of 2SpamH), respectively, on users with a grid of phone usage and activity levels. The simulation parameters $L_{A}$ and $L_{C}$ vary from 0.3 to 0.8 with a step size of 0.1. We define two performance measures, sensitivity, that is, the proportion of correctly identified missing observations, and specificity, that is, the proportion of correctly identified non-missing observations. For each user, 1000 datasets each with 100 observations, where each observation is composed of the variables number of uploads (UP), number of screen unlocks (SU), number of device notifications (DN), and battery variance or BV are simulated as described in Section 2.5 and the Supplementary Materials. The heatmaps in Figure 4 display sensitivity and specificity results for the three methods, zero-removal, one-class SVM, and 2SpamH, across different activity levels and phone-usage levels. Each heatmap represents a different method, with rows indicating activity levels ($L_{A}$) and columns indicating phone-usage levels ($L_{C}$). The sensitivity and the specificity are calculated on the 1000 measurement matrices of the user for the specific $L_{A}$ and $L_{C}$ using the labels inferred by 2SpamH and the true labels.

2SpamH exhibits a consistently high sensitivity across various phone-usage and activity levels, ranging from 0.66 to 0.77. This robustness in identifying missing observations, regardless of user behavior patterns, is likely due to its user-level training. On the other hand, the one-class SVM shows a wide range of sensitivity values, from as low as 0.14 to as high as 0.90, suggesting that the method’s ability to detect missing observations is highly dependent on the specific combination of phone-usage and activity levels, performing best at higher activity levels. Zero-removal demonstrates intermediate sensitivity, generally higher than one-class SVM but still lower and more variable than 2SpamH, with values between 0.05 and 0.73. Its performance improves with increased activity and phone-usage levels.

While one-class SVM and zero-removal excel in detecting non-missing observations, showing improving specificity with higher phone-usage and activity levels, respectively, their performances come at the cost of highly variable sensitivity. 2SpamH’s occasional poor specificity, where “non-missing” observations are misclassified as “missing”, can be addressed by adjusting the threshold for prototype selection. This adaptability is an advantage over methods like one-class SVM, which cannot be applied to real data since the model is trained on data known to be “non-missing”, as this is unknown in real life. Notably, in the context of our problem, high sensitivity is particularly crucial because it ensures that most unreliable and missing observations are correctly identified, thereby maintaining the integrity and completeness of passive data. In Supplementary Figure S1, we present the sensitivities and specificities for 2SpamH using different values of the thresholds (i.e., $\theta_{lower}=\left(0.20,\;0.40\right)$ and $\theta_{upper}=\left(0.60,\;0.80\right)$). This figure demonstrates that the threshold can be used to increase sensitivity at the cost of specificity (e.g., when using a (0.20, 0.80) threshold) or increase specificity at the cost of sensitivity (e.g., when using a (0.40, 0.60) threshold).

### 3.2. Real-Life mHealth Dataset Results

**Figure 5:**
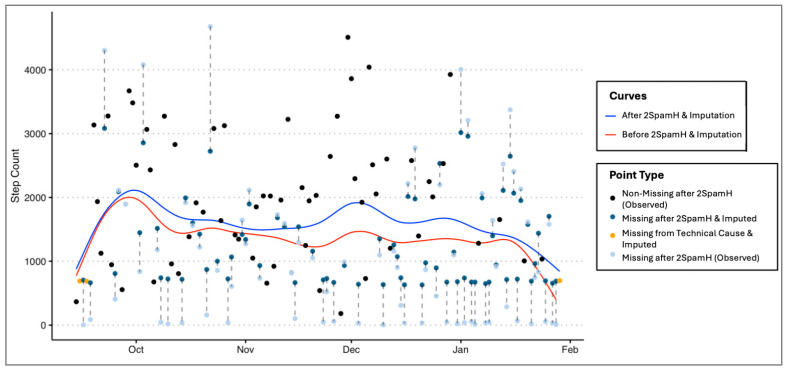
Trajectory of step count data before and after applying 2SpamH and imputation. Data points are color-coded to differentiate their types: black (Point Type: Non-Missing after 2SpamH (Observed) dots represent the values of step counts of good quality observations identified by the 2SpamH algorithm; turquoise (Point Type: Missing after 2SpamH and Imputed) dots represent the values of step counts of poor-quality observations identified by the 2SpamH algorithm, which were then imputed using missForest; orange (Point Type: Missing from Technical Cause and Imputed) dots represent missing data points in the original data and then imputed using missForest; and gray (Point Type: Missing after 2SpamH (Observed)) dots represent the values of the observed step counts in the original data of poor-quality observations identified by 2SpamH. Note that each gray dot is connected to a turquoise dot by a dashed line since these are identified as missing by the 2SpamH algorithm and imputed. The blue curve shows the trend of the step counts after applying the 2SpamH algorithm and imputing missing data, while the red curve shows the trend before applying the algorithm. The figure demonstrates the effectiveness of 2SpamH in addressing the underestimation problem in passive measures.

To demonstrate the performance of 2SpamH, we utilized smartphone data collected in research studies conducted at the Weill Cornell ALACRITY Center. The Center’s studies involved adults aged 55 and older with major depressive disorder undergoing psychotherapeutic interventions. A smartphone app collected various passive sensing data, phone usage metrics, and sensor activity measures. In Figure 5 below, we present the example of one user illustrating that 2SpamH can potentially address the problem of downward bias in passive measures. A smoothed trajectory (blue curve) of the post-processed step counts after applying 2SpamH and imputation lies above the trajectory fit on the un-processed step counts (red curve), indicating there is likely an underestimation in the unprocessed data, which if ignored can lead to incorrect conclusions about trajectories and trends in the passive data. As noted earlier, this is important because trajectories and trends are the only meaningful information that can be obtained from passive data recorded on commercial devices.

**Figure 6:**
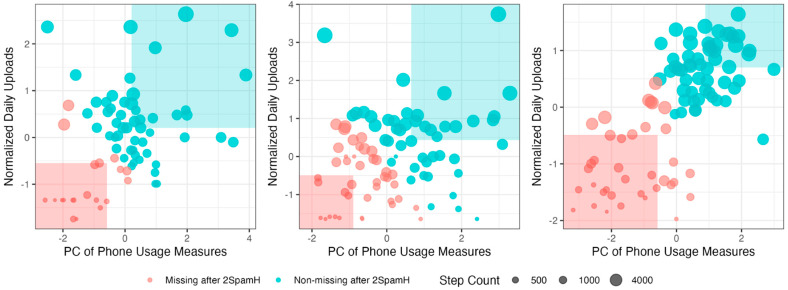
2SpamH algorithm for three users. Each point represents a daily observation of step count, the *x*-axis represents the first principal component of phone usage measures, and the *y*-axis represents the normalized number of uploads. The red-shaded areas in the lower left corners and blue-shaded areas in the upper right corners of each subplot represent prototypes with missing and non-missing labels, respectively. The size of the dots corresponds to the number of steps, with larger dots indicating higher step counts.

Figure 6 below demonstrates the labeling of step count observations for three users after applying the 2SpamH algorithm. Figure 6 demonstrates how the thresholds can select a different number of prototypes for each user. Depending on the distributions of a participant’s device use and sensor activity, different quantities of a passive variable will be labeled as “missing”.

### 3.3. Imputation Results

Table 2 presents the normalized root mean squared error (NRMSE) between the observed and imputed data in our imputation simulation for several passive variables. Across all passive variables except for conversation percentage, both versions of missForest had a lower NRMSE compared to jomoImpute (Table 2). Moreover, missForest applied on all 106 users jointly had a lower NRMSE compared to the same when applied to each user separately except for the conversation percentage and total activity duration. This suggests that the imputation model learns patterns across users. Therefore, missForest applied on all users jointly is our recommended method for imputing 2SpamH-labeled missing data.

## 4. Discussion and Conclusions

In this study, we proposed the 2SpamH algorithm, an unsupervised two-stage algorithm designed to address the downward bias introduced by the non-use or non-wear of mHealth devices. As the device use or wear is unknown to the data analyst, 2SpamH infers device use for each user-day (or any other time interval) in two stages: first, it selects prototypes where device use can be assumed to be known with some confidence; and second, it infers device-use status for non-prototypes with KNN. Device non-use or non-wear are then treated as missing data which are then imputed using various off-the-shelf methods like missForest and jomoImpute. Consequently, this pipeline of pre-processing yields a clean passive sensing dataset that can be used for downstream analysis of within-user behavioral trajectories and that has accounted for the downward bias introduced due to device non-use or non-wear. Our simulations demonstrate that 2SpamH has a superior performance in correctly identifying “non-missing” labels compared to zero-removal or one-class SVM. Real-life data from a clinical study further validate the algorithm’s ability to handle passive data effectively by correcting the downward bias, ensuring data integrity for downstream analysis.

Despite the promise of 2SpamH, it is not without limitations, particularly regarding parameter tuning. The selection of thresholds for prototype selection in Stage 1 and the number of nearest neighbors (*k*) in Stage 2 are subjective. As noted, while the algorithm performs well with the 30th and 70th percentiles for threshold selection, these thresholds may need to be adjusted based on the specific dataset. The need for manual tuning of these parameters introduces subjectivity and limits the algorithm’s scalability. Although we recommended strategies to choose these parameters based on the dataset size and distribution, future iterations of the algorithm could benefit from an automated tuning mechanism. We have shown in our simulations that modifying the thresholds such that there are fewer prototypes selected will improve sensitivity (correctly identifying non-missing data) and reduce specificity (correctly identifying missing data), while modifying the thresholds such that there are more prototypes selected will increase specificity while reducing sensitivity. Therefore, tuning adjustments need to be made for each user depending on the context. Another limitation of 2SpamH is that it evaluates device usage and sensor activity one user at a time to personalize the pre-processing pipeline; however, relationships between device usage and sensor activity can be learned across users which could potentially improve the detection of device non-use. We recommend future work to improve the performance of 2SpamH that borrows information across users.

In conclusion, the 2SpamH algorithm addresses a critical gap in the pre-processing of passive sensing data by identifying device non-use or non-wear. The downward bias in passive data introduced by the device non-use or non-wear of commercial devices can significantly distort user-specific patterns of passive measures over time. 2SpamH’s personalized approach to identify device non-use or non-wear in each user as missing data and to impute missing data enables downstream analysis and makes passive data from commercial devices usable. This is particularly important in healthcare applications where missing or unreliable data can obscure user behavior and health outcomes. Without such pre-processing, studies may under-utilize the potential of passive sensing data. We have also developed a freely available software, implemented in R, that can help scientists to use and analyze passive sensing data [26]. As passive sensing becomes more prevalent in healthcare, algorithms like 2SpamH will be essential for ensuring data quality and enabling the use of passive measures to their fullest potential.

## Figures and Tables

**Table 1:** Suggested labels given phone-usage level and activity level and their real-life implications.

Device Usage	Number of Uploads	Implication	Prototype Label
High	High	Engaging with the device	Non-Missing
High	Low	Using the device but inactive	N/A
Low	High	Active, carrying/wearing the device but not engaging	N/A
Low	Low	Not engaged and not carrying/wearing the device	Missing

**Table 2:** NRMSE results for the passive measures for each imputation technique.

Measure	MissForest (Within-User)	JomoImpute	MissForest (Across Users)
Step Count	0.44	0.44	0.31
Time at Home	0.39	0.54	0.32
Conversation Percentage	0.14	0.18	0.19
Time in Conversation	0.24	0.30	0.24
Sleep Duration	0.43	0.57	0.37
Travel Diameter	0.59	0.88	0.44
Active Time	0.37	0.52	0.27
Sleep Interruptions	0.53	0.68	0.23
Radius of Gyration	0.46	0.83	0.33
Total Activity Duration	0.36	4.46	0.41
Total Location Duration	0.42	0.52	0.26

## Data Availability

Data will be made available upon request.

## Supplementary Materials

The following supporting information can be downloaded at https://www.mdpi.com/article/10.3390/s24217053/s1, Figure S1: Simulation results under different thresholds; Figure S2: Real data results under different thresholds.
