# Supplementary material for: 2SpamH: A Two-Stage Pre-Processing Algorithm for Passively Sensed mHealth Data
Source: Sensors (Basel). 2024 Oct 31;24(21):7053. doi: 10.3390/s24217053 (PMC11548539; doi:10.3390/s24217053)
Supplement: Supplementary file 1 [file sensors-24-07053-s001.zip › sensors-3183347-supplementary.pdf]

# SUPPLEMENTARY FILES

## Details of Simulation Studies

This section provides the detailed simulation strategy used in Section 2.5 to mimic real-world data. We simulated data to compare the performance of 2SpamH with the zero-removal technique and the one-class support vector machine [16].

Passive sensing data is simulated for 36 users daily for 100 days to demonstrate the effect of 2SpamH on a passive variable, e.g., step count. All users have 24 observations simulated per day representing each hour of the day. For each hour, battery levels, number of uploads, screen unlocks, and device notifications were simulated as device usage variables. The hourly observations are then aggregated at the daily level by calculating the sum of the hourly data for all variables except battery variance, for which variance is calculated. Each hourly observation is randomly assigned binary activity and device use status $\{\boldsymbol{A_h}, \boldsymbol{U_h}\}$ with the subscript $h$ representing the hour of the day. The status $\boldsymbol{A_h}$ represents user physical activity level and corresponds with the amount of device uploads, with $\boldsymbol{A_h = 1}$ meaning that the user is active, and uploads are recorded, and $\boldsymbol{A_h = 0}$ meaning that the user is inactive and few uploads are recorded. Note that, the activity variable $\boldsymbol{A_h}$ is assumed to indicate both sensor activity and user activity (i.e., step counts) synonymously. Similarly $\boldsymbol{U_h = 1}$ corresponds to high device use and $\boldsymbol{U_h = 0}$ corresponds to low device use. Using the $\{\boldsymbol{A_h}, \boldsymbol{U_h}\}$ hourly labels, the daily ground truth is "non-missing" when both $\boldsymbol{A_h = 1}$ and $\boldsymbol{U_h = 1}$, and "missing" otherwise. We denote these daily, ground truth labels as $\boldsymbol{M_t^*}$.

The hourly binary status $\{\boldsymbol{A_h}, \boldsymbol{U_h}\}$ are generated from Bernoulli distributions with probabilities $\boldsymbol{p_A^h(L_A)}$ and $\boldsymbol{p_C^h(L_U)}$ where $\boldsymbol{h \in \{1, 2, \dots 24\}}$. Note that the probability distribution change hourly depending on the time of day, e.g., probabilities are higher in the during the morning and afternoon, lower in the evenings, and they become 0 during the night (see definitions below). The probabilities of high activity and device use are also generated randomly from a uniform distribution which depends on two parameters, $\boldsymbol{L_A}$ and $\boldsymbol{L_U}$, that represent the continuous levels of activity and device usage. Higher $\boldsymbol{L_A}$ and $\boldsymbol{L_U}$ correspond to higher probabilities and consequently higher frequency of 1 in the $\{\boldsymbol{A_h}, \boldsymbol{U_h}\}$. During the morning and afternoon hours, the Bernoulli probabilities are generated from $\boldsymbol{Uniform(L_A, L_A + 0.2)}$ representing an activity level $\boldsymbol{L_A}$ or higher, while during the evening the probabilities are generated from $\boldsymbol{Uniform(L_A - 0.2, L_A)}$ representing an activity level $\boldsymbol{L_A}$ or lower and are exactly 0 at night. Details of the Bernoulli probabilities are stated below.

$$\boldsymbol{h \in \{1, \cdots, 24\}}$$

$$\boldsymbol{Morning\ (m) = \{6, \cdots, 12\}, Afternoon\ (a) = \{13, \cdots, 17\},}$$

$$\boldsymbol{Evening\ (e) = \{18, \cdots, 21\}, Night\ (n) = \{1, \cdots 5, 22, \dots, 24\}}$$

$$\boldsymbol{p_A^h(L_A) = \begin{cases} U(L_A, L_A + 0.2) & if\ h \in \{m, a\} \\ U(L_A - 0.2,\ L_A) & if\ h \in e \\ 0 & if\ h \in n \end{cases}}$$

$$\boldsymbol{p_U^h(L_U) = \begin{cases} U(L_U,\ L_U + 0.2) & if\ h \in \{m, a, e\} \\ 0 & if\ h \in n \end{cases}}$$

*Simulation of uploads (UP)*

Sensory activity is operationalized by the total daily uploads of a given sensor (see Section 2.1 of main text). Total daily uploads for a given day *t* for a user ($\boldsymbol{UP_t}$) is the sum of active, hourly observations

$$\boldsymbol{UP_t = \Sigma_{h=1}^{24} I(A_h = 1) I(U_h = 1)}$$

where $\boldsymbol{I}(.)$ is the indicator function.

*Simulation of screen unlocks (SU)*

Screen unlocks ($\boldsymbol{SU_h}$) are simulated from a Poisson distribution with rate parameter $\boldsymbol{\mu_h}$, and $\boldsymbol{\mu_h}$ is set to 0 when the device is not used ($\boldsymbol{U_h = 0}$). Otherwise, if the user was active ($\boldsymbol{A_h = 1}$), unlocks are less frequent, and we set $\boldsymbol{\mu_h = 3}$. If the user was not active ($\boldsymbol{A_h = 0}$), we set $\boldsymbol{\mu_h = 20}$. For day *t*, the daily screen unlocks is $\boldsymbol{SU_t = \Sigma_{h=1}^{24} SU_h}$.

*Simulation of device notifications (DN) sent by the mHealth app that are viewed*

Notifications ($\boldsymbol{DN_h}$) sent by the mHealth app and viewed by the user is an indication of user engagement with the device. $\boldsymbol{DN_h}$ events are also simulated using a Poisson distribution with rate parameter $\boldsymbol{\lambda_h}$. Users are simulated to receive on average 5 notifications per hour ($\boldsymbol{\lambda_h = 5}$) in the morning and afternoon ($\boldsymbol{m, a}$), and on average 1 notification per hour ($\boldsymbol{\lambda_h = 1}$) in the evening ($\boldsymbol{e}$). No notifications ($\boldsymbol{\lambda_h = 0}$) were generated at night ($\boldsymbol{n}$). For day *t*, the daily device notification is $\boldsymbol{DN_t = \Sigma_{h=1}^{24} DN_h}$.

*Simulation of battery variance (BV)*

Hourly battery levels ($\boldsymbol{B_h}$) were simulated between 0% and 100%. At night ($\boldsymbol{n}$), the battery is assumed to be fully charged. During the day, battery drain occurred at a constant rate, adjusted for screen unlocks and device notifications:

$$\boldsymbol{B_h = B_{h-1} - \frac{1}{24} - (c_1 \times SU_h + c_2 \times DN_h)}$$

Here, we assume there is a constant battery drain of 1/24 per hour, and we use $\boldsymbol{c_1 = 5 \times 10^{-4}, c_2 = 1 \times 10^{-4}}$ to model the battery consumptions of screen unlocks and device notifications, respectively. Charging occurs with a probability positively correlated to device engagement and inversely related to battery level, modeled as $\boldsymbol{c_1 p_U^h(L_U) \times (1 - B_h)}$. Recall $\boldsymbol{p_U^h(L_U)}$ is the Bernoulli probability for $\boldsymbol{U_h}$. Daily battery variance ($\boldsymbol{BV_t}$) is calculated as the variance of $\boldsymbol{B_h}$ for day *t*.

Data on each of the 36 users are simulated for a period of 100 days. Each row of sensor activity data matrix $\boldsymbol{W}$ contains the daily summations of uploads ($\boldsymbol{UP_t}$), while each row in device usage matrices $\boldsymbol{Z}$ contain the screen unlocks ($\boldsymbol{SU_t}$), device notifications ($\boldsymbol{DN_t}$) and battery variances ($\boldsymbol{BV_t}$). $\boldsymbol{W, Z}$ are the input for the 2SpamH algorithm (Algorithm 1) separately for each simulated user. Note that the passive variable matrix, $\boldsymbol{X}$, is not directly used by 2SpamH and only used in the imputation stage. We use the 2SpamH Stage 1 thresholds of {30, 70} and k = 5 for the KNN in Stage 2. The output labels $\boldsymbol{M_t}$ from 2SpamH are compared against the ground truth labels $\boldsymbol{M_t^*}$. We generated 1000 datasets using this structure and used two types of performance metrics, namely sensitivity and specificity, to demonstrate the performance of 2SpamH. The sensitivity is defined as the proportion of "non-missing" in $\boldsymbol{M_t^*}$ that are correctly identified by $\boldsymbol{M_t}$, and the specificity is defined as the proportion of "missing" in $\boldsymbol{M_t^*}$ that are correctly identified by $\boldsymbol{M_t}$. Both of the metrics are reported in Figure 5 across ranges of the continuous levels of activity $\boldsymbol{L_A}$ and device usage $\boldsymbol{L_C}$.

**Supplemental Figures**

**A**

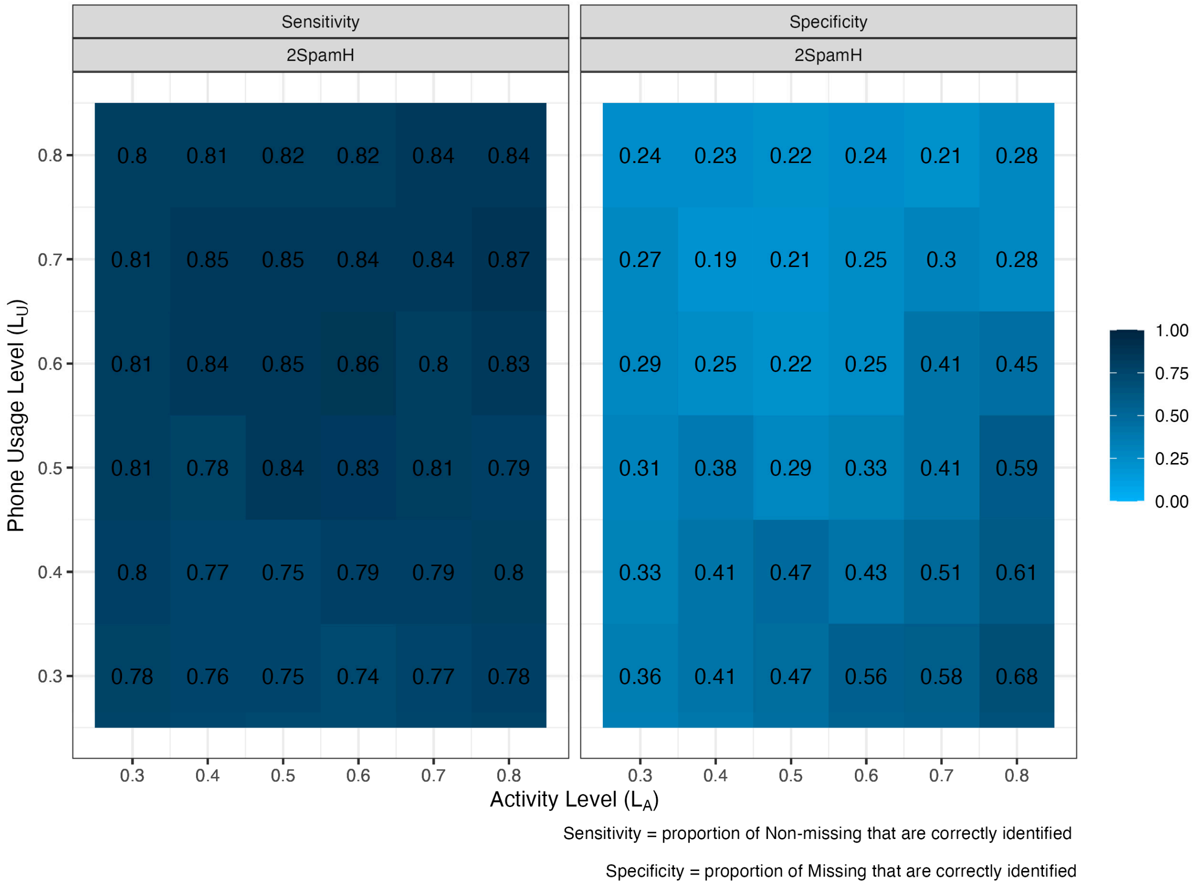
Sensitivity
2SpamH
Specificity
2SpamH
Phone Usage Level ($L_U$)
Activity Level ($L_A$)
1.00
0.75
0.50
0.25
0.00
Sensitivity = proportion of Non-missing that are correctly identified
Specificity = proportion of Missing that are correctly identified

**B**

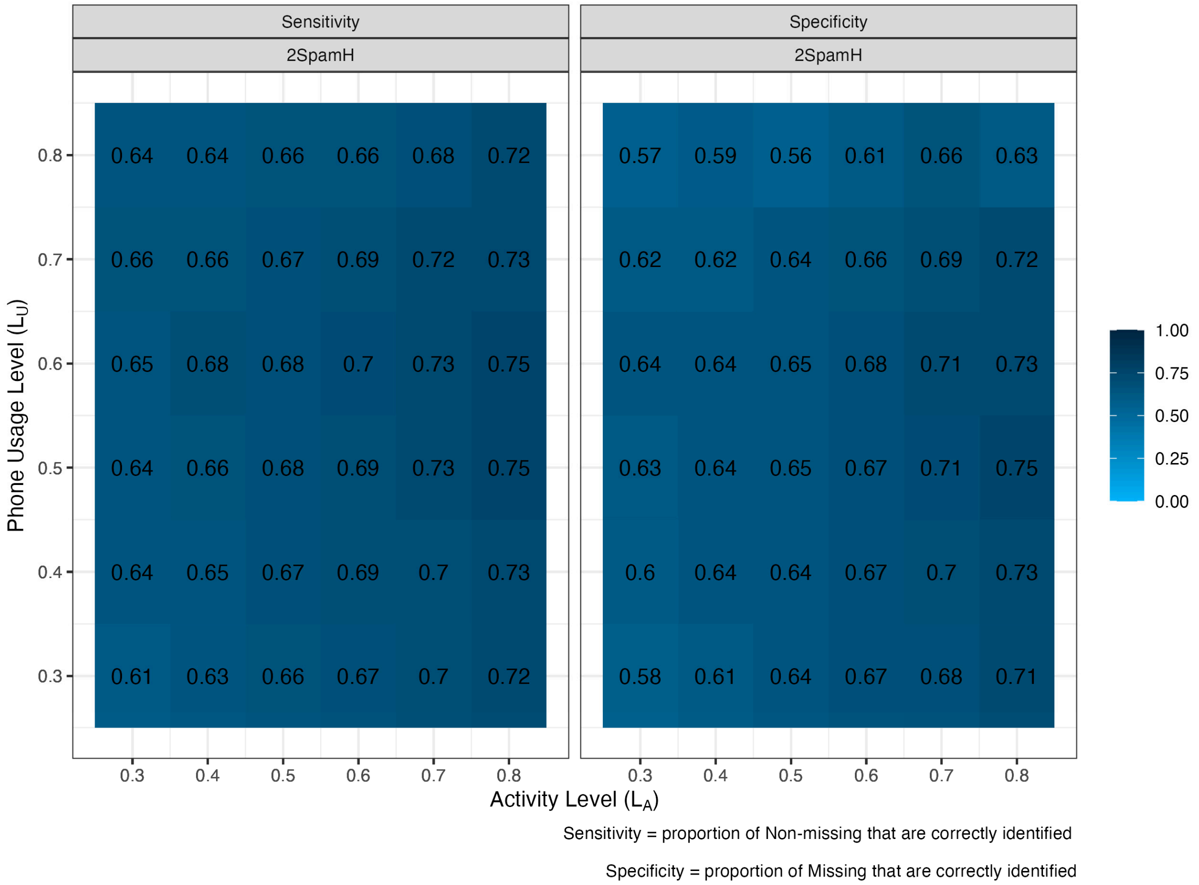


**Supplementary Figure S1.** Simulation results under different threshold values, 20th and 80th quantiles (Panel A) and 40th and 60th quantiles (Panel B).

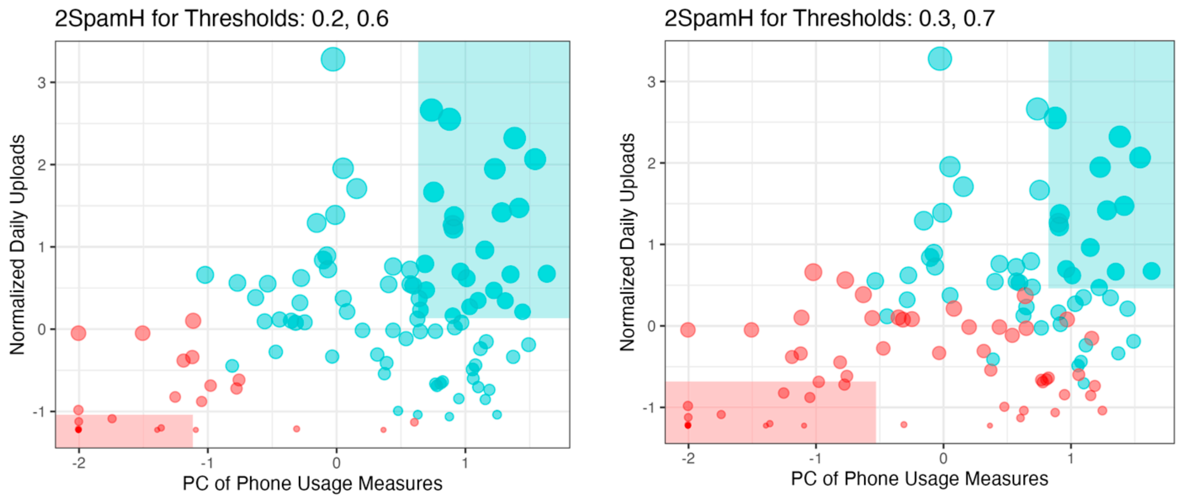

**Supplementary Figure S2:** 2SpamH for different thresholds on the same user. Each point represents a daily observation of step count, the x-axis represents the first principal component of phone usage measures, and the y-axis represents the normalized number of uploads. The size of the dots corresponds to the number of steps, with larger dots indicating higher step counts. The red-shaded areas in the lower left corners and blue-shaded areas in the upper right corners of each subplot represent prototypes with "missing" and "non-missing" labels, respectively. Red step counts are labeled as "missing" after 2SpamH while blue step counts are labeled as "non-missing."

**Algorithm Validation with Authorization Data**

In addition to passive sensing data, some mHealth technologies also automatically log user authorizations for the sensors, which potentially provides a set of quality labels for the 2SpamH algorithm. When a sensor is misconfigured, for example when the user sets the permission to "only while the app is open'' rather than "always allow" or the user has completely denied the permission for data collection, all measures derived from this sensor mimic patterns like when device usage is low to none. Ideally, these variables should be labeled as "missing" by the 2SpamH algorithm. Using the GPS sensor as an example, there are fourteen days in total for all users the sensor was reported as misconfigured. We applied the 2SpamH for all observations from each user, and these fourteen days are all labeled as "missing." The results provide additional real-data evidence for 2SpamH's capability to identify faulty observations.
